# Why not? Understanding the spatial clustering of private facility-based delivery and financial reasons for homebirths in Nigeria

**DOI:** 10.1186/s12913-018-3225-4

**Published:** 2018-06-01

**Authors:** Kerry L. M. Wong, Emma Radovich, Onikepe O. Owolabi, Oona M. R. Campbell, Oliver J. Brady, Caroline A. Lynch, Lenka Benova

**Affiliations:** 1 Department of Infectious Disease Epidemiology, Faculty of Epidemiology and Population Health, London School of Hygiene and Tropical Medicine, Keppel Street, London, WC1E 7HT UK; 2 Guttmacher Institute, 125 Maiden Lane 7th Floor, New York, NY 10038 USA; 3 Centre for Mathematical Modelling for Infectious Diseases, London School of Hygiene and Tropical Medicine, Keppel Street, London, WC1E 7HT UK

**Keywords:** Spatial epidemiology, Clustering, Facility childbirth delivery, Maternal health service utilization, Financial barrier, Private health services

## Abstract

**Background:**

In Nigeria, the provision of public and private healthcare vary geographically, contributing to variations in one’s healthcare surroundings across space. Facility-based delivery (FBD) is also spatially heterogeneous. Levels of FBD and private FBD are significantly lower for women in certain south-eastern and northern regions. The potential influence of childbirth services frequented by the community on individual’s barriers to healthcare utilization is under-studied, possibly due to the lack of suitable data.

Using individual-level data, we present a novel analytical approach to examine the relationship between women’s reasons for homebirth and community-level, health-seeking surroundings. We aim to assess the extent to which cost or finance acts as a barrier for FBD across geographic areas with varying levels of private FBD in Nigeria.

**Method:**

The most recent live births of 20,467 women were georeferenced to 889 locations in the 2013 Nigeria Demographic and Health Survey. Using these locations as the analytical unit, spatial clusters of high/low private FBD were detected with Kulldorff statistics in the SatScan software package. We then obtained the predicted percentages of women who self-reported financial reasons for homebirth from an adjusted generalized linear model for these clusters.

**Results:**

Overall private FBD was 13.6% (95%CI = 11.9,15.5). We found ten clusters of low private FBD (average level: 0.8, 95%CI = 0.8,0.8) and seven clusters of high private FBD (average level: 37.9, 95%CI = 37.6,38.2). Clusters of low private FBD were primarily located in the north, and the Bayelsa and Cross River States. Financial barrier was associated with high private FBD at the cluster level – 10% increase in private FBD was associated with + 1.94% (95%CI = 1.69,2.18) in nonusers citing cost as a reason for homebirth.

**Conclusions:**

In communities where private FBD is common, women who stay home for childbirth might have mild increased difficulties in gaining effective access to public care, or face an overriding preference to use private services, among other potential factors. The analytical approach presented in this study enables further research of the differentials in individuals’ reasons for service non-uptake across varying contexts of healthcare surroundings. This will help better devise context-specific strategies to improve health service utilization in resource-scarce settings.

**Electronic supplementary material:**

The online version of this article (10.1186/s12913-018-3225-4) contains supplementary material, which is available to authorized users.

## Background

Despite ongoing efforts by the Nigerian health system to increase maternal health service utilization, including midwives service schemes, removal of user fees and increasing the involvement of the private sector [1, 2], population usage of many life-saving obstetric interventions remains suboptimal. National statistics for 2009–2013 show that, for instance, 22.6% of all births occurred in a public health facility and 13.2% in the private sector – leaving approximately two thirds of childbirths based outside of a health facility [3]. At the subnational scale, likelihoods for facility-based delivery (FBD) and private FBD vary considerably, and were significantly lower for women residing in parts of the South South zone, and majority of the Northern zone [3].

Both in Nigeria and other low- and middle-income countries (LMICs), having a FBD is a practical way to ensure assistance by a skilled birth attendant and access to life-saving interventions for mothers and newborn [4]. Previous reviews addressing factors related to FBD in sub-Saharan Africa and other LMICs have identified an array of determinants [4–7]. Moyer and Mustafa’s literature review, published in 2013, highlighted an overwhelming reliance on population/survey data with which maternal sociodemographic factors were well-represented [4]. The limited body of literature around community-level factors of FBD in LMICs emphasizes community socio-demographic characteristics, community views on skilled and traditional births [8, 9], service accessibility such as distance to care and community uptake of antenatal care [4]. Communities likely have other unique characteristics that influence demand for and supply of healthcare [10], many of which are overlooked.

Unlike other health service seeking, childbirth can happen unexpectedly throughout the day and the woman may need to reach a nearby care provider at relatively short notice. The types of childbirth delivery services most accessible to, or most accessed by, the community directly relate to an individual’s perception of, wishes for, and actual uptake of services. Women also exchange information and experience surrounding childbirth in social settings, and one’s planning for future delivery may be conditioned by assessing factors important to their peers, culture and community [11, 12]. A better understanding of one’s healthcare surroundings is imperative to developing effective strategies to increase healthcare utilization among groups currently “left behind”. Part of the dearth of research in this area might be due to the lack of suitable data, especially at the national scale.

In a study of the characteristics of health facilities across Nigeria, Nwakeze and Kandala found vast geographic disparities in the country, including greater dominations of lower-level and primary care and private health services in some areas but not others [13]. In addition, despite the Nigerian government’s aspiration to provide free/subsidized maternity care in the public sector, some women who stay home for childbirth reported cost or finance as a barrier to using maternity care, among other factors [14, 15]. This raises questions regarding current understanding of the factors for service uptake vs. non-uptake in relation to one’s healthcare surroundings. In some settings, e.g. where public maternity care is free of charge, it is likely that some of those who stay home for childbirth for financial reasons only considered using private services, the alternative being homebirths (over public care). This speculation might be more pertinent where private FBD is common, due to the potential impact that one’s peers and healthcare surroundings have on their reasons for service non-uptake.

The aim of this study is to assess the extent to which cost or finance is a barrier for FBD across geographic areas with varying levels of private FBD in Nigeria. To overcome the limitation of community-level data availability, we present an innovative approach applying geographic information system (GIS) tools to examine the clustering of maternity care utilization using individual-level survey data. This study will help motivate and enable further investigation of the way in which childbirth services frequented by the community influences community members to deliver in or outside a health facility, adding contribution to the current effort to support maternity care utilization for groups and individuals most “left behind”.

## Methods

### Data and study sample

This analysis was based on data from the 2013 Nigeria Demographic and Health Survey (NDHS). The data is representative at the national level, of the six geopolitical zones and of the 36 states and the Federal Capital Territory (FCT-Abuja). The survey sample was selected using a stratified multi-stage cluster probability sampling design with census ward as the primary sampling unit. As part of the DHS sampling procedure, all households in each sampled ward was enlisted, which was then used as the sampling frame for household selection [16]. Eligible individuals aged 15–49 in selected households were interviewed with a standardized questionnaire. The final sample of the 2013 NDHS consisted 896 census wards and 39,902 eligible women; 98% (38,948) were successfully interviewed. Women with a live birth in the five years before the interview were asked to self-report the care received during pregnancy and delivery. The sample of the current analysis was restricted to the circumstances of 20,467 women’s most recent live birth during the five-year survey recall period as some of the required data was only collected for this subsample.

### Geography and administration of Nigeria

**Fig. 1:**
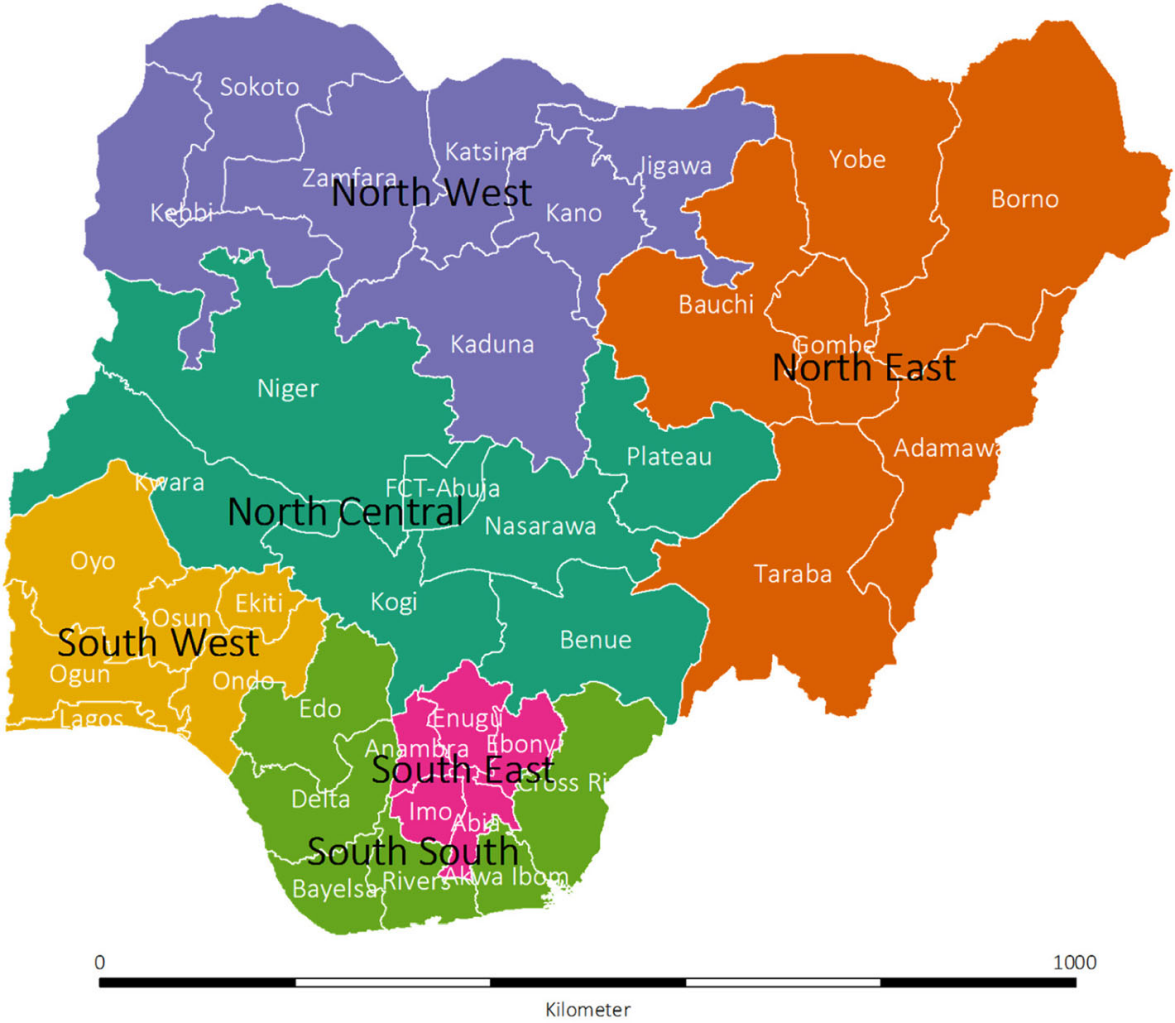
Map of Nigeria showing boundaries of six geopolitical zones, 36 states and Federal Capital Territory (FCT-Abuja). Shapefile is obtained from gadm.org. The 2018 GADM license allows data re-use for academic and other non-commercial purposes (https://gadm.org/license.html, last accessed: 14th May 2018)

Nigeria is divided into six geopolitical zones (Fig. 1): North Central, North East, North West, South West, South South and South West; and within these zones, into 36 states and the FCT-Abuja. For administrative purposes, the states are subdivided into 774 local governments areas [3], each made up of approximately 10–15 wards [17].

### Measurement

Population centroids of wards, recorded as latitude and longitude, were obtained by DHS enumerators using Global Position System (GPS) receivers [18]. All individuals residing in a ward therefore have the same georeference. For privacy considerations, the coordinates were randomly displaced by up to 2 km in urban areas and up to 5 km in rural areas by the NDHS. An additional 1% of rural wards were displaced by 10 km.

Delivery location was based on women’s answer to: “Where did you give birth to [name of child]?” on the Women’s Questionnaire. The major categories of response were domestic environments (home of respondent or of traditional birth assistant (TBA)), public or governmental health facilities (HFs), private or non-governmental HFs, as well as all other unspecified locations [3]. FBD was obtained by coding responses as “any HF” and “not in a HF”. For the analysis of private FBD, all births were categorized as “any private HF” and “not in a private HF”. We note that the 2013 NDHS had conflated all non-governmental, for-profit and not-for-profit providers as one category of “private” provider.

The outcome of interest was financial barrier for FBD. Women who did not deliver in a HF indicated the reasons that applied to them from a list of potential barriers, including “cost too much”. Other covariates and demographic information considered as potential confounders were: wealth quintile, maternal education, maternal age and parity at the time of the most recent birth, and whether the woman had health insurance coverage. Household wealth quintile was derived from the wealth index – constructed by the DHS using household asset data via a principal component analysis [19]. The sampled households were than ranked and divided into five quintiles. Each woman is assigned her household’s wealth quintile.

### Spatial scan statistics of private FBD

To identify geographic clusters of high and low private FBD, the number of most recent births and those based in a private HF were aggregated at the ward level, with adjustment of survey sampling weighting. Together with ward latitude and longitude as inputs, each ward was treated as an analytical unit to test whether private FBDs were distributed randomly in space or not.

At the ward level, the observed numbers of private FBD varied from zero to the total number of eligible births. To detect clustering of private FBDs, we chose a Poisson distribution to represent the expected distribution of this count over space. Under the null hypothesis, the expected number of private FBDs in each area is proportional to its population size (approximated using sample size) [20, 21]. Spatial scan statistics was performed using the SaTScan™ software (version 9.4) [20, 21]. Spatial clusters were identified by taking into consideration the rates of nearby wards [22, 23]. The spatial scan method used circular windows of various sizes that move across the map to find clusters of wards with either higher and lower than expected rates under the null hypothesis of uniform spatial distribution [24, 25]. The radius of the circle varies continuously from zero to a predefined value that specified the percentage of the maximum total population at risk within the scanning window [21]. The recommended maximum size is 50%; we conducted additional scans at the 10 and 5% levels to account for independent smaller clusters that may be contained in a large cluster. The alternative hypothesis is that there is a reduced/elevated rate within the scanning window as compared to outside. The test of significance, based on likelihood ratio and the null distribution, was obtained from Monte Carlo Simulation [26]. The number of permutations was set at 999 and the significance level was set at 0.05 [21]. Identified clusters are ordered based on their likelihood ratio test values.

Geographic locations of, and wards contained in each, identified spatial cluster were merged back to the 2013 NDHS women’s data. We considered women living in the same SaTScan spatial cluster to be in the same “community”. Estimates on private service use as a percentage of all most recent births, financial barriers reported among women who did not deliver in a HF, as well as other covariates were recalculated for each SaTScan spatial cluster to generate community-level data. This was done in Stata SE version 14 (StataCorp LP, College Station, TX, USA), adjusted for survey-specific weighting and stratified, cluster sampling design.

### Relating community -level private facility use to nonusers’ self-reporting of financial barrier

Using SaTScan spatial cluster as the analytical unit, the percentage of nonusers reporting financial barrier (main outcome) was related to the percentage of births occurring in private facilities. The SatScan spatial clusters were weighted by the number of most recent births circled within. To account for a proportion as outcome (bounded between 0 and 100%), we adopted a generalized linear model, specifying a logit link and the binomial family [27–29]:

$$ \mathrm{logit}\left({\mathrm{p}}_{\mathrm{i}}\right)=\mathrm{x}\upbeta +{\upvarepsilon}_{\mathrm{i}}\mathrm{where}\ {\mathrm{Y}}_{\mathrm{i}}\sim \mathrm{Bin}\left({\mathrm{N}}_{\mathrm{i}},{\mathrm{p}}_{\mathrm{i}}\right),{\upvarepsilon}_{\mathrm{i}}\sim \mathrm{N}\left(0,{\upsigma}^2\right) $$

We denoted *y*_*i*_ = number of private FBD in SaTScan spatial cluster *i*, *N*_*i*_ = number of most recent births in *i* and *p*_*i*_ = probability of having a private FBD. We also specified the Huber-White (i.e. robust) estimators of the standard errors in case of heteroskedasticity arising from potential misspecification in the distribution family [30]. The z test was used for significance testing of model coefficients. We generated predictions from both the bivariate and multivariate fits and back-transformed them as the percentages of women with a non-facility birth who cited cost was a barrier at 5%-intervals of community-level private FBD.

### Missing data

We found missing data in geographic coordinates in seven wards, containing < 1% of the respondents from the study sample. These were removed from analyses where location data was required. We also found 0.4% of missing data for health insurance coverage and coded these as uninsured. There was no missing data in the other variables in the model.

## Results

### Facility-based delivery

Of the 20,467 births in our sample, 7649 (37.4, 95%CI = 34.7,40.2) occurred in health facilities: 23.8% (95%CI = 22.0,25.5) in public and 13.6% (95%CI = 11.9,15.5) in private facilities. More of those who were rural residents, from the poorest wealth quintile, without any education, uninsured and having a second or higher order birth delivered outside of a health facility (Table 1). Geographic variations of FBD were observed – highest in the South East zone (78.5, 95%CI = 73.2,83.0) and lowest in the North West zone (12.8, 95%CI = 10.2,15.9).

**Table 1:** Percentage distribution and 95% confidence intervals of sample sociodemographic characteristics by place of delivery

		Number of most recent births	Place of delivery		
			Outside of a health facility	Public health facility	Private Health facility
N proportion		20,467	12,818	5100	2620
		(100)	62.6 (59.8,65.3)	23.8 (22.0,25.6)	13.6 (11.9,15.5)
Area of residence	Urban	6790	36.8 (32.6,41.2)	36.3 (33.5,36.3)	26.9 (23.2,30.8)
	Rural	13,402	76.9 (74.2,79.4)	16.8 (15.0,18.8)	6.3 (5.2,7.7)
Wealth quintile	Poorest	4379	93.8 (92.4,95.0)	5.0 (4.1,6.1)	1.2 (0.8,1.9)
	Poorer	4603	81.8 (79.2,84.1)	13.4 (11.7,15.3)	4.8 (3.7,6.2)
	Middle	4069	62.2 (58.7,65.5)	26.6 (24.0,29.3)	11.3 (9.4,13.4)
	Richer	3798	42.5 (38.9,46.1)	39.4 (36.5,42.3)	18.2 (15.7,20.9)
	Richest	3343	18.6 (16.2,21.3)	42.5 (38.7,46.3)	38.9 (34.3,43.7)
Maternal education	No education	9171	88.0 (86.4,89.5)	10.2 (8.9,11.6)	1.8 (1.4,2.3)
	Primary	4113	57.1 (54.0,60.3)	27.5 (25.2,29.9)	15.4 (13.4,17.6)
	Secondary	5565	33.8 (31.1,36.5)	39.0 (36.4,41.6)	27.2 (24.0,30.7)
	Higher	1343	8.3 (8.3,10.7)	51.3 (46.7,55.9)	40.4 (35.5,45.5)
Health insurance	Yes	363	14.7 (10.4,20.4)	50.6 (43.8,57.4)	34.7 (27.8,42.3)
	No	19,829	63.4 (60.6,66.1)	23.3 (21.6,25.1)	13.3 (11.6,15.1)
Parity	First birth	3624	51.7 (48.3,55.0)	31.4 (28.9,34.0)	16.9 (14.6,19.5)
	Higher order birth(s)	16,568	65.0 (62.2,67.7)	22.1 (20.4,23.9)	12.9 (11.3,14.7)
Geopolitical zones	North Central	3095	53.0 (47.4,58.5)	31.3 (27.7,35.2)	15.7 (12.7,19.3)
	North East	4001	79.5 (74.9,83.4)	19.2 (15.5,23.5)	1.3 (0.8,2.1)
	North West	6206	87.2 (84.1,89.8)	12.3 (9.8,15.2)	0.5 (0.3,1.1)
	South East	1724	21.5 (17.0,26.8)	33.7 (28.9,38.9)	44.8 (38.4,51.4)
	South South	2500	49.2 (44.1,54.4)	36.6 (32.6,40.7)	14.2 (10.8,18.3)
	South West	2666	23.8 (19.3,29.0)	23.8 (31.9,40.6)	40.1 (35.2,45.1)
Age at birth	Mean (interquartile range)	29.42 (29.2,29.6)			
Self-reported financial barrier to deliver in a health facility			9.1 (8.9,10.5)	*Not applicable*	*Not applicable*

### Sub-national private facility-based delivery

Regional averages of private FBD varied between 0.5% (95%CI = 0.3,1.1) in North West zone to 44.8% (95%CI = 38.4,51.4) in the South East zone (Table 1). Using SaTScan analysis, ten spatial clusters of low level and seven spatial clusters of high private FBD were identified (Table 2). The number of wards contained in these geographic clusters ranged from five to 88; the number of most recent births circled within a geographic cluster ranged between 63 and 1201, and spatial cluster radii varied between 21.2 and 208.5 km. Altogether, 648 wards and 14,434 births occurred in these 17 clusters.

**Table 2:** Seventeen significantly higher and lower than expected proportions of FBD spatial clusters

	ID	Cluster location			No. of wards circled	No. of most recent births	Observed number of private FBD	Observed % private FBD	Expected number of private FBD under H_0_	Relative risk^+^	*p*-value^$^
		Latitude	Longitude	Radius (km)							
High	1^^^	6.7	3.7	97.2	75	1182	605	52.8	154	4.82	< 0.001
	2	9.9	8.9	21.2	5	63	33	50.5	8	4.06	< 0.001
	3	5.8	7.2	85.2	88	1199	569	48.9	156	4.38	< 0.001
	4	6.7	5.4	78.7	28	457	146	32.9	60	2.54	< 0.001
	5	8.5	4.6	132.5	67	1198	338	28.0	156	2.34	< 0.001
	6	7.3	9.0	79.4	12	249	73	27.4	32	2.29	< 0.001
	7	7.9	7.1	148.3	78	1200	295	25.3	156	2.00	< 0.001
Low	8	9.8	9.7	64.0	8	233	8	3.1	30	0.26	0.014
	9	5.3	8.6	75.9	17	280	8	2.9	37	0.22	< 0.001
	10	4.6	5.7	88.6	24	558	5	2.1	73	0.07	< 0.001
	11	8.7	11.6	178.0	41	1187	22	1.8	155	0.14	< 0.001
	12	10.8	7.3	155.6	38	1174	14	1.4	153	0.09	< 0.001
	13	10.8	3.9	184.8	24	770	7	0.5	100	0.07	< 0.001
	14	13.3	8.0	144.9	31	1201	1	0.1	156	0.01	< 0.001
	15	12.0	12.5	208.5	46	1197	2	0.1	156	0.01	< 0.001
	16	11.7	9.3	84.7	30	1085	1	0.1	141	0.01	< 0.001
	17	13.2	5.5	145.6	36	1201	0	0.0	156	0.00	< 0.001

FBD = facility based delivery; H_0_ = null hypothesis of spatial randomness

^$^The likelihood ratio test is used for testing cluster significance

^^^Cluster 1 is the most likely cluster; all other clusters are non-overlapping secondary clusters

^+^Relative risk of private FBD within cluster compared to the risk in all other areas

**Fig. 2:**
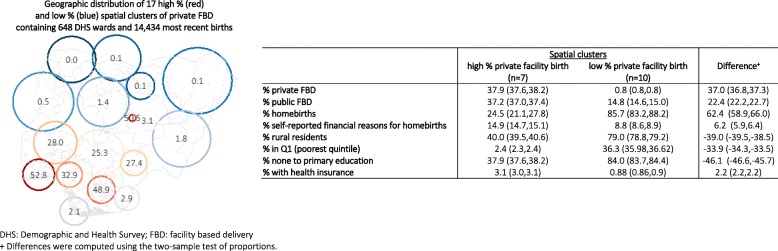
Seventeen SaTScan spatial clusters (drawn proportionate to cluster radii) of higher (red) and lower (blue) than expected proportions of private facility birth among all most recent births. The DHS wards contained in each spatial clusters are also shown

The location and size of these geographic clusters were drawn in Fig. 2. Clusters of low private FBD were primarily located in the North West and North East zones, with an exception near Jos North in Plateau State, where one spatial cluster of high private FBD (50.5, 95%CI = 35.5,65.5) was identified. In addition, southern Cross River state and central and southern Bayelsa state (in the South South zone) also showed spatial clustering of low private FBD: 2.9% (95%CI = 1.0,4.7) and 2.1% (95%CI = 0.0,5.5), respectively. Communities of high private FBD were identified around the Lagos and Ogun States (52.8, 95%CI = 47.7,57.9), Edo State (32.9, 95%CI = 24.7,41.1) as well as large parts of the South-East zone (e.g., Imo and Abia States) and the North Central zone.

Mean percentages of private FBD in high and low clusters were 37.9% (95%CI = 37.6,38.2) and 0.8% (95%CI = 0.8,0.8), respectively. Average public FBD among all births was 37.2% (95%CI = 37.1,37.4) in high private FBD clusters. On the other hand, 14.8% of all births were public facility-based in the ten spatial clusters of low private FBD. Substantial differences in sociodemographic characteristics of women living in the two groups of spatial clusters were also seen (Fig. 2). Women in low private FBD clusters were more rural, poorer and less educated compared to women in high private FBD clusters.

We performed additional cluster detections setting maximum cluster size to 10 and 5% of the survey sample. The first yielded the same set of results. The details of the 19 SaTScan spatial clusters returned from using the 5% limit is given in Additional file 1: Figure S1. No substantial differences to the model with 17 SatScan clusters were observed.

### Reporting cost as a barrier for facility-based delivery

**Fig. 3:**
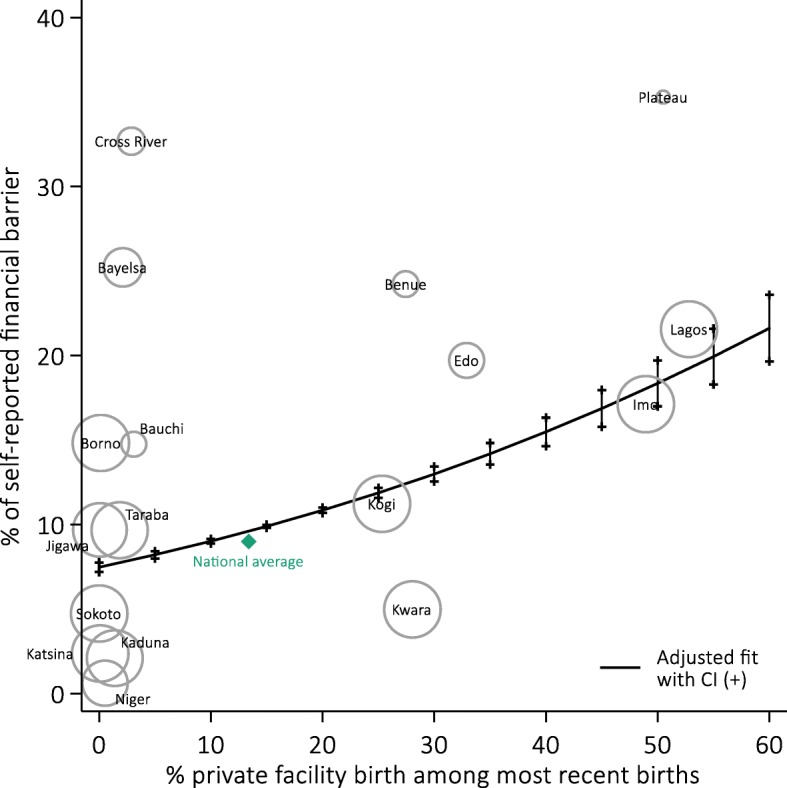
Proportions of women delivering outside a health facility who self-reported financial barrier as a reason for homebirth in 17 spatial clusters of high and low private facility births. Predicted percentages and confidence intervals at various levels of private facility birth from an adjusted generalized linear model weighted by numbers of most recent births in spatial clusters are also shown (represented by size of bubbles)

Across the seven spatial clusters of high private FBD, 24.5% (95%CI = 21.1,27.8) of women delivered at home and 14.9% (95%CI = 14.7,15.1) reported cost as a barrier (Fig. 2). In contrast, 85.7% (95%CI = 83.2,88.2) of women living in the 10 clusters with low FBD delivered at home, and 8.8% (95%CI = 8.6,8.9) cited cost as a barrier. Figure 3 illustrates that in contrast to other spatial clusters of low private FBD, exceptionally high proportions of nonusers living in Cross River (32.7, 95%CI = 26.3,39.1) and Bayelsa State (25.2, 95%CI = 19.1,31.4) said cost was a reason to deliver outside a facility. Unadjusted analysis showed that the factors associated with self-reported financial barrier for FBD at the spatial cluster unit level included living in Cross River and Bayelsa States, the percentage of public facility utilization, rural setting, wealth, the level of maternal education, and the percentage of women covered by health insurance (Table 3). All of these were significant at the *p* < 0.001 level.

**Table 3:** Effect sizes of predictor variables and estimates^^^ of proportion citing financial barriers

Community-level factors	Unadjusted estimates		Adjusted estimates	
	Average change in proportion of nonusers citing financial barriers with 95%CI and p-value			
Private facility delivery (every + 10%)	1.82 (1.79,1.86)	< 0.001	1.94 (1.69,2.18)	< 0.001
Public facility delivery (every + 10%)	1.17 (1.14,1.20)	< 0.001	−1.64 (−1.88,-1.41)	< 0.001
Rural sample (every + 10%)	−1.20 (−1.24,-1.16)	< 0.001	0.66 (0.54,0.78)	< 0.001
Wealth: Q1 sample (every + 10%)	−2.32 (2.37,2.27)	< 0.001	0.50 (0.31,0.70)	< 0.001
No to primary education (every + 10%)	−1.83 (−1.86,-1.80)	< 0.001	− 1.61 (− 1.78,-1.43)	< 0.001
Health insurance (every + 10%)	21.8 (21.0,22.7)	< 0.001	3.58 (2.11,5.05)	< 0.001
Geographic location				
Others	Reference		Reference	
Cross River and Bayelsa	17.61 (17.34,17.87)	< 0.001	17.34 (15.74,18.94)	< 0.001
% of births in private facility	Predicted percentage of nonusers citing financial barriers as reason for homebirth with 95%CI^+^*			
0	8.23 (8.08,8.37)		7.48 (7.20,7.76)	
5	8.96 (8.82,9.10)		8.22 (8.00,8.43)	
10	9.75 (9.62,9.89)		9.02 (8.88,9.16)	
15	10.60 (10.48,10.73)		9.90 (9.81,9.99)	
20	11.52 (11.40,11.64)		10.85 (10.69,11.01)	
25	12.51 (12.40,12.62)		11.88 (11.59,12.17)	
30	13.57 (13.46,13.68)		12.99 (12.55,13.44)	
35	14.70 (14.60,14.81)		14.19 (13.56,14.83)	
40	15.92 (15.80,16.02)		15.49 (14.64,16.33)	
45	17.21 (17.08,17.33)		16.87 (15.79,17.96)	
50	18.58 (18.42,18.73)		18.36 (17.00,19.71)	
55	20.03 (19.85,20.22)		19.94 (18.29,21.59)	
60	21.57 (21.35,21.80)		21.62 (19.66,23.59)	

^ Unadjusted and adjusted effects were back-transformed from parameter estimates obtained using a logit link transformation. The z test was used for significance testing of model coefficients

+ Adjusted estimates describe the adjusted curve drawn in Fig. 4

*Adjusted predicted percentage of nonusers citing financial barriers were obtained with all other coverages fixed their mean values

In multivariate analysis, all predictors remained significantly associated with the proportion of women reporting financial barrier in the community (Table 3). After controlling for the proportion of birth occurring in public HFs, rurality, wealth, maternal education, health insurance and residency in Cross River and Bayelsa States, a 10% point increase in private facility use for childbirth was associated with an average 1.94% point increase (95%CI = 1.69,2.18) in nonusers citing cost as a barrier for FBD. The adjusted predicted percentages of self-reported financial barrier across varying levels of private service use were also computed based on the adjusted regression model. Table 3 and Fig. 3 illustrate a steady rise in the extent to which financial consideration was a barrier as community-level private FBD increased.

## Discussion

To our knowledge, this is the first study to examine national geographic disparities in private facility use for childbirth in a sub-Saharan African country at a small geographic scale. We found substantial spatial variation in the utilization of private facilities for delivery care across Nigeria. The level of private FBD was very low in the northern part of the country except for Jos in Plateau State. Private FBD was medium to high in North Central zone and the highest in the South West and South East zones. Certain areas in Lagos, Imo, Ogun and Abia States had particularly high levels of private FBD. Using a novel approach, we examined the association between private healthcare utilization contexts and financial barriers for FBD. We found cost was more likely to be cited as a barrier to FBD in settings where private FBD was high. We found exceptions, however, for southern Cross River and Bayelsa States, where a large proportion of nonusers reported cost as a barrier and overall facility delivery (in both the public and private sectors) very low.

### Limitations

Our findings have important implications, but they should be understood with certain limitations. Firstly, the 2013 NDHS response option for private delivery included both for-profit and not-for-profit establishments operating under different financial motives and potentially charging widely varying fees for childbirth care. However, we still believe that our assumption that private sector childbirth costs more than public sector is valid. Self-reported reasons to deliver in non-healthcare settings might also be subject to accuracy and reliability issues [31]. In addition, women could list more than one barrier of FBD – approximately 50% of women who cited cost as a barrier also listed one or more other reasons (data not shown) – and the relative importance of cost compared to other reasons is not known. Contributions of other potential factors – including, but not limited to individuals’ perceptions towards the care received and healthcare professionals – warrants further investigation. The analytical approach presented in this study offers a novel method for such future research with available, secondary data.

The SaTScan spatial clusters identified were relatively large in geographical size (even with a smaller maximum allowable limit), and there might be substantial heterogeneity in the characteristics of the women living in the same spatial cluster. Some of this heterogeneity, including parity, pregnancy complication and marital status, may confound our primary association of interest at the individual-level, but were omitted as their relevance at the community level is likely low. Lastly, some loss of power in cluster detection might have occurred through a degradation of spatial information between the exact geographic coordinates of individuals and those at aggregated levels [32, 33].

### Giving birth in the private sector

In Nigeria and other LMICs, pregnant women who opt for private FBD have a similar sociodemographic profile – higher SES, higher education and, in some contexts, certain ethnicity or religious affiliations [34–37]. A search of peer-reviewed articles and the grey literature returned little information on the cost of private FBD in Nigeria. However, a study showed 1.8 times more spending in private hospitals than public hospitals by users residing in urban south-eastern Nigeria [38]. Despite higher cost, for-profit healthcare care may have more appeal due to a wide range of reasons, such as privacy, shorter waiting times, higher perceived quality of care, empathy and respectful approach, availability of doctors and as a status symbol [39, 40]. For users of private services, cost or affordability might be a relatively weaker determinant of where to seek care.

### Community-level private service use and self-reported financial barriers for facility-based delivery

Our findings extend the current knowledge about preference towards private HFs for their users. We found that in contexts with relatively high private FBD, a greater proportion of facility non-users reported financial barriers for any care, including both private care and the relatively more affordable public care. In Edo, Ogun and Abia States, for instance, the majority of health facilities are privately owned [13]. Our results may indicate that facility nonusers living in high FBD contexts are unable to gain effective access to any healthcare due to personal financial barrier (for private care) and insufficient provision of public services in their lived environment. In other places of high private FBD where such practice may have become normalized, women who lack adequate funds for private providers might perceive delivering at home or a TBA’s home as their best alternative due to social pressure and low acceptability of publicly provided services. The observed preference for homebirths is in line with qualitative findings from various states including FCT-Abuja and Lagos, where women who do not deliver in a health facility had poor confidence in the public health sector and strong desires to deliver with a TBA [41–43]. According to these studies, women perceive home delivery with a TBA, and especially with family members present, to be personal and supporting [41]. Some TBAs often allow for flexible finance options, such as payment in kind or in instalments, making it easier for families to pay [42].

On the other hand, in settings where private facility delivery use is relatively low, and especially where overall FBD utilization is also low, such as most of North West zone and North East zone, women’s reasons to not give birth in a HF were less connected to cost. In these settings, other cited barriers included service availability, distance or physical accessibility, social norms and lack of perceived need [43]. In a study set in the Jigawa State, approximately 25% of nonusers claimed they did not attend facilities for childbirth because they did not think it was necessary [44]. In addition, household decision-making dynamics also varies across this large multi-ethnic country; Abuja city/FCT-Abuja, for instance, is generally associated with greater gender equality when compared to other southern and northern cities [45]. Especially in the north, women’s relative lack of participation in intra-household decision making and access to money have been associated with very low FBD rates [45].

Exceptions to the inverse relationship found between financial barriers and private FBD were noted in southern Cross River State and Bayelsa State, where overall percentages of FBD were midrange, private FBD very low, and a relatively large proportion of nonusers reported financial barriers to delivering in a HF. This highlights the importance of contextualizing personal factors alongside other community- or macro-level factors. Bayelsa State is primarily covered by marshlands and waterways; it is also an important gas- and petrol-producing region in Nigeria that has generated interest among prospective companies [46, 47]. However, most Bayelsans remain poor, and the state’s public infrastructure development insufficient [47]. Lack of transportation and the riverine setting pose tremendous impediments to overcoming physical barriers to reaching health services [46, 48]. In a study looking at barriers to utilization of maternal health services in Bayelsa State, a majority of respondents reported infrastructure-related barriers to access (availability of facilities/equipment, schedule of maternal health clinic, accessibility and so on); and much lower percentages of women reported deterrents such as cultural acceptance and language problems [49]. Compared to the rest of the country, special economic and environmental contexts and the additional resources required to overcome physical accessibility barriers may have caused financial considerations to operate differently among people living in Bayelsa and Cross River States. The role of financial barriers, separating direct payment for delivery from other expenses and trade-offs, including cost of transport, as well as time and financial lost from other daily/productive activities, warrants further research.

### A note on using DHS data to study healthcare utilization surroundings

Various studies have looked at the service provision environment as a determinant of FBD. A common approach consists of conducting interviews with women about the availability of maternity care in their community as a measure of service provision [50–54]. Alternatively, geocoded master facility list (MFL) data or the like, with which the entire health infrastructure of a spatial area is mapped out, are geographically linked to population data in a GIS to facilitate calculation of measures of people’s healthcare availability [55–57]. The present study used available secondary data on individual-level service utilization and women’s location of residence to construct the geographic patterning of healthcare surroundings across Nigeria. Our variable of interest was community-level utilization surrounding the individuals, which is somewhat conditioned on healthcare provision environment, but is also a consequence of other cultural, contextual and individual-level determinants. Nwakeze and Kandala examined the spatial distribution of health establishments using data collected by the National Bureau of Statistics of Nigeria, and found moderate to low numbers of private health establishments in the Benue, Nasarawa and Kogi States, compared to the number of public health facilities [13]. In the present analysis, however, parts of these places showed high level of private FBD. Our findings therefore also tangentially shed light on people’s decision-making of the services to use from the options that are available to them. Such knowledge is useful for the formulation of appropriate interventions to concurrently address provision of and demand for services [58, 59]. In the case of these states, additional provision of public health services might not be as effective a strategy to boost FBD as trying to strengthen the quality and acceptability of existing public services.

## Conclusion

In this study, we found an inverse relationship between community private care-seeking for childbirth and self-reported financial reasons of service non-uptake. This extends current understanding of the influence of financial barriers for maternity care. We argue that further investigation of determinants of maternal health-seeking, and potentially other health-seeking, should look beyond individual-level barriers to consider community-level factors. Many LMICs continue to be challenged by poor maternal health outcomes driven to some extent by wide subnational disparities in maternal healthcare provision, utilization and care quality. The lack of research and attention in the existing literature to study community-level factor is possibly due to the lack of suitable data, especially since studies of determinants of FBD are mostly based on individual- and household-level data. Working with geographic data and GIS tools, including mapping techniques and spatial cluster detection, we developed a novel way to bridge this persistent knowledge gap. Our approach offers new approaches to examine the way in which childbirth services frequented by the community influences community members to deliver in or outside a health facility. The method presented can be extended to other research questions related to barriers and different health service characteristics, such as service acceptability and the level/standard of care most frequently sought, as well as perceived need, cultural drivers and social norms against overall utilization rate. Our approach also preserves spatial patterns in the data, a component that is often neglected but requires specific analytical considerations and carries contextual significance, including policy implications.

Overall, we suggest that the approach presented to be best for 1) illustrating the service utilization environment in the population and 2) examining associations between individual-level and community-level factors. The complex reasons behind underutilization of delivery care services indicates the need for a multi-focus approach that addresses service provision and usage suited for the local context of healthcare uptake and non-uptake. Further research is needed to help inform policies and health system responses to provide adequate health services that people will utilize.

## Additional file

Additional file 1: **Figure S1.** Nineteen SaTScan spatial clusters (drawn proportionate to cluster radii) of higher and lower than expected proportions of private facility birth among all most recent births. The DHS wards contained in each spatial clusters are also shown. (DOCX 130 kb)
