# Supplementary material for: Why not? Understanding the spatial clustering of private facility-based delivery and financial reasons for homebirths in Nigeria
Source: BMC Health Serv Res. 2018 Jun 1;18:397. doi: 10.1186/s12913-018-3225-4 (PMC5984741; doi:10.1186/s12913-018-3225-4)
Supplement: Supplementary file 1 — Figure S1. Nineteen SaTScan spatial clusters (drawn proportionate to cluster radii) of higher and lower than expected proportions of private facility birth among all most recent births. The DHS wards contained in each spatial clusters are also shown. (DOCX 130 kb) [file 12913_2018_3225_MOESM1_ESM.docx]

**Additional file 1 Figure S1** Nineteen SaTScan spatial clusters (drawn proportionate to cluster radii) of higher and lower than expected proportions of private facility birth among all most recent births. The DHS wards contained in each spatial clusters are also shown.

|  | **ID** | **Cluster location** | | | **No. of**  **wards**  **circled** | **No. of**  **most recent births** | **Observed number of**  **private FBD** | **Observed % private FBD** | **Expected**  **number of private FBD**  **under H_0_** | **Relative**  **risk^+^** | **p-value^$^** |
| --- | --- | --- | --- | --- | --- | --- | --- | --- | --- | --- | --- |
|  |  | **Latitude** | **Longitude** | **Radius (km)** |  |  |  |  |  |  |  |
| **High** | 1^^^ | 9.9 | 8.9 | 21.2 | 5 | 63 | 33 | 52.4 | 8 | 4.06 | <0.001 |
|  | 2 | 6.6 | 3.3 | 101.7 | 63 | 984 | 512 | 52.0 | 128 | 4.73 | <0.001 |
|  | 3 | 6.0 | 7.1 | 76.9 | 74 | 999 | 473 | 47.3 | 130 | 4.22 | <0.001 |
|  | 4 | 7.9 | 4.5 | 84.9 | 61 | 997 | 323 | 32.4 | 130 | 2.70 | <0.001 |
|  | 5 | 6.7 | 5.4 | 78.7 | 28 | 457 | 146 | 31.9 | 60 | 2.54 | <0.001 |
|  | 6 | 4.5 | 7.3 | 71.0 | 27 | 384 | 121 | 31.5 | 50 | 2.49 | <0.001 |
|  | 7 | 7.3 | 9.0 | 79.4 | 12 | 249 | 73 | 29.3 | 32 | 2.29 | <0.001 |
|  | 8 | 7.9 | 7.1 | 137.8 | 63 | 954 | 235 | 24.6 | 124 | 1.98 | <0.001 |
| **Low** | 9 | 9.8 | 9.7 | 64.0 | 8 | 233 | 8 | 3.4 | 30 | 0.26 | 0.011 |
|  | 10 | 8.0 | 11.0 | 147.7 | 18 | 587 | 20 | 3.4 | 76 | 0.26 | <0.001 |
|  | 11 | 5.0 | 8.3 | 43.7 | 14 | 230 | 4 | 1.7 | 30 | 0.13 | <0.001 |
|  | 12 | 11.0 | 8.1 | 108.4 | 32 | 999 | 10 | 1.0 | 130 | 0.07 | <0.001 |
|  | 13 | 10.6 | 5.5 | 173.3 | 29 | 870 | 8 | 0.9 | 113 | 0.07 | <0.001 |
|  | 14 | 10.5 | 11.1 | 120.7 | 33 | 999 | 9 | 0.9 | 130 | 0.07 | <0.001 |
|  | 15 | 4.6 | 5.7 | 88.6 | 24 | 558 | 5 | 0.9 | 73 | 0.07 | <0.001 |
|  | 16 | 12.7 | 13.6 | 300.5 | 39 | 1001 | 2 | 0.2 | 130 | 0.02 | <0.001 |
|  | 17 | 12.2 | 9.3 | 80.7 | 27 | 1001 | 1 | 0.1 | 130 | 0.01 | <0.001 |
|  | 18 | 12.6 | 6.6 | 107.8 | 27 | 1001 | 0 | 0.0 | 130 | 0.00 | <0.001 |
|  | 19 | 12.9 | 4.7 | 114.1 | 30 | 994 | 0 | 0.0 | 129 | 0.00 | <0.001 |

^­­­­­­­^FBD = facility based delivery; H_0_ = null hypothesis of spatial randomness

^$^ The likelihood ratio test is used for testing cluster significance.

^^^ Cluster 1 is the most likely cluster; all other clusters are non-overlapping secondary clusters.

^+^ Relative risk of private FBD within cluster compared to the risk in all other areas.
